# Pre-Emptive Endoluminal Negative Pressure Therapy at the Anastomotic Site in Minimally Invasive Transthoracic Esophagectomy (the preSPONGE Trial): Study Protocol for a Multicenter Randomized Controlled Trial

**DOI:** 10.29337/ijsp.24

**Published:** 2021-03-18

**Authors:** Philip C. Müller, Diana Vetter, Joshua R. Kapp, Christoph Gubler, Bernhard Morell, Dimitri A. Raptis, Christian A. Gutschow

**Affiliations:** 1 Department of Visceral und Transplant Surgery, University Hospital Zurich, Rämistrasse 100, 8091 Zürich, Switzerland; 2 Department of Hepatology and Gastroenterology, University Hospital Zurich, Rämistrasse 100, 8091 Zürich, Switzerland; 3 Department of Surgery, Royal Free Hospital, Pond Street London NW3 2QG, United Kingdom

**Keywords:** Anastomotic leakage, endoluminal vacuum therapy, randomised controlled trial, minimally invasive oesophagectomy

## Abstract

**Introduction:**

Anastomotic leakage (AL) accounts for a significant proportion of morbidity following oesophagectomy. Endoluminal negative pressure (ENP) therapy via a specifically designed polyurethane foam (EsoSponge^®^, B.Braun Medical, Melsungen, Germany) has become the standard of care for AL in many specialized centres. The prophylactic (pENP) application of this technique aims to reduce postoperative morbidity and is a novel approach which has not yet been investigated in a prospective study. The aim of this study is therefore to assess the effect of pENP at the anastomotic site in high-risk patients undergoing minimally invasive transthoracic Ivor Lewis oesophagectomy.

**Methods and analysis:**

The study design is a prospective, multi-centre, two-arm, parallel-group, randomised controlled trial and will be conducted in two phases. Phase one is a randomised feasibility and safety pilot trial involving 40 consecutive patients. After definitive sample size calculation, additional patients will be included accordingly during phase two. The primary outcome of the study will be the postoperative length of hospitalization until reaching previously defined “fit for discharge criteria”. Secondary outcomes will include postoperative morbidity, mortality and postoperative AL-rates based on 90-day follow-up. A confirmatory analysis based on intention-to-treat will be performed.

**Ethics and dissemination:**

The ethics committee of the University of Zurich approved this study (2019-00562), which has been registered with *ClinicalTrials.gov* on 14.11.2019 (NCT04162860) and the Swiss National Clinical Trials Portal (SNCTP000003524). The results of the study will be published and presented at appropriate conferences.

## 1. Background

Anastomotic leakage (AL) is a life-threatening complication following visceral surgery. AL occurs in 5–30% of patients after esophagectomy and may be further complicated by mediastinitis, sepsis, multiple organ failure, or death [1,2,3]. Despite extensive research in animal models and humans, the biological process of anastomotic healing and the exact causes of AL are largely unknown. A recent hypothesis is that the pathophysiology of this entity involves a triad of local ischaemia, bacterial infection, and inflammation [4,5,6]. Furthermore, predisposing factors such as arteriosclerosis, diabetes, and lung disease have been associated as risk factors for the development of AL in patients undergoing oesophagectomy [7,8,9,10].

Endoscopic placement of a specially designed polyurethane foam at the level of the anastomosis with subsequent endoluminal negative pressure (ENP) therapy has a high success rate in treating AL [11,12,13]. The negative pressure continuously removes wound secretions, improving interstitial oedema and microcirculation, thus inducing tissue granulation which results in accelerated healing of the infected area. Moreover, a recent case series demonstrated that prophylactic ENP (pENP) in patients with proven local ischemia at the anastomosis was effective in preventing AL in six of eight patients. [14] However, in this study, the ENP device was placed during the early postoperative course after anastomotic ischemia was detected, whilst the present study evaluates a prophylactic application of ENP in patients identified to be high risk for developing AL. Recently, we have published a retrospective series of patients undergoing minimally invasive oesophagectomy with pENP therapy. Anastomotic healing was uneventful in 19/20 anastomoses resulting in a low AL rate of 5%. The single case of AL in these series successfully healed without further complications in response to a second course of ENP therapy. No adverse events attributable to pENP therapy were observed in this series [15].

The aim of the present study is to assess the effect of pENP placement at the anastomotic site on postoperative outcome following minimally invasive transthoracic oesophagectomy, with a focus on postoperative length of hospital stay and morbidity. The study is designed to address the following hypothesis: pENP therapy may reduce postoperative length of hospital stay and morbidity in high-risk patients undergoing total minimally invasive (laparoscopic and thoracoscopic) transthoracic Ivor Lewis esophagectomy (ttMILE).

## 2. Methods and analysis

The objective of this initiative is to assess the effect of pENP therapy in high-risk patients undergoing ttMILE on postoperative length of hospital stay, morbidity and mortality. The study has been reported in line with the Consolidated Standards of Reporting Trials (CONSORT) criteria [16].

The following hypotheses will be tested:

H0: Patients undergoing pENP after ttMILE have similar postoperative morbidity and hospital stay compared with patients who do not undergo pENP.

H1: Patients undergoing pENP after ttMILE have a lower postoperative morbidity and a shorter hospital stay compared with patients who do not undergo pENP.

### 2.1. Registration

This trial has been registered with *ClinicalTrials.gov*, a primary registry in the WHO, on 14.11.2019 under the registration number NCT04162860 and with the Swiss National Clinical Trials Portal [*www.kofam.ch*] under the registration number SNCTP000003524.

### 2.2. Study design

**Figure 1:**
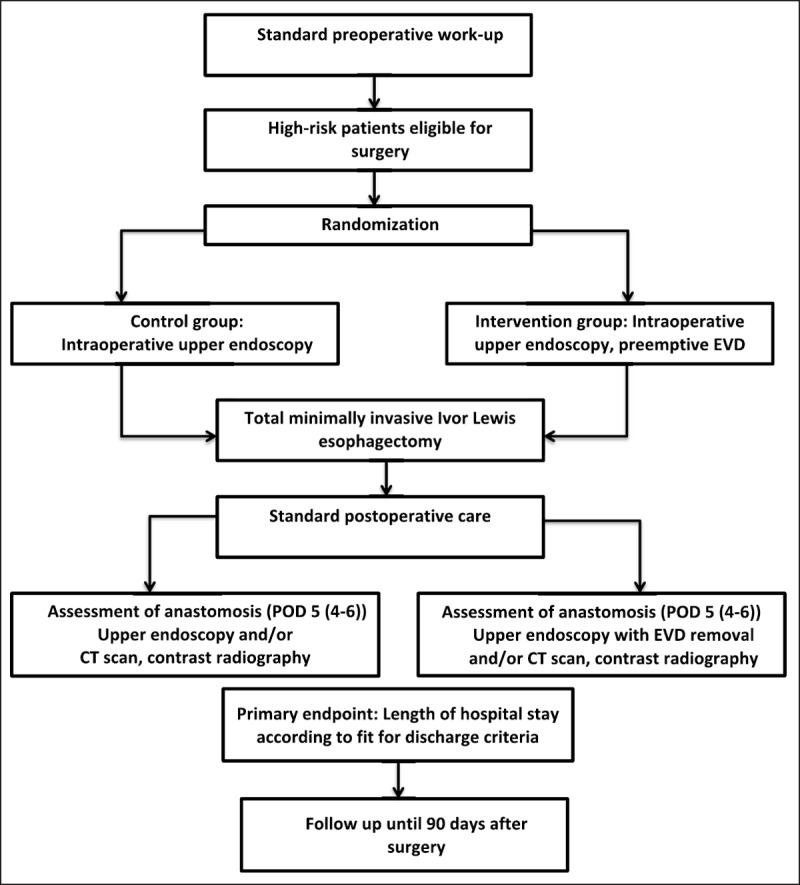
Study flow chart of the randomized preSPONGE trial.

This study is a prospective multi-centre, two-arm, parallel-group randomised controlled trial. The study flow chart is shown in ***Figure 1***.

### 2.3. Study setting

The trial will be led by the Department of Visceral und Transplant Surgery, University Hospital Zurich, Zurich, Switzerland. The University Hospital Zurich is a high-volume expert centre for oesophageal surgery with an annual caseload of around 40 esophagectomies. In addition, we intend to recruit 3–4 additional European centres of similar caseload.

### 2.4. Sample size determination

Since no data is available on which sample size calculation could be based, a randomized pilot feasibility and safety study including 40 consecutive patients is planned.

For the completion of the formal preSponge RCT, additional patients will be included after definitive sample size calculation which will take place following inclusion of the first 40 patients in the feasibility study. It is anticipated that a total of 100 patients (approximately 50 per group) will be needed.

### 2.5. Inclusion and exclusion criteria

We will include adult high-risk patients (≥18 years of age) who provided informed consent and are scheduled for ttMILE. Robotic-assisted procedures will also be included.

Patients with **at least one of the following risk factors** will be considered at high risk for developing AL:

- ASA score >2
- Diabetes (insulin dependent or HbA1c ≥ 6.5%)
- Chronic pulmonary disease (FEV1/FVC ratio ≤ 70%)
- Heart failure (LVEF <55%)
- Pre-existing cardiac arrhythmia (pacemaker or paroxysmal supraventricular tachyarrhythmia)
- Chronic kidney disease stage 4–5 (GFR < 30ml/min/1.73 m^2^)
- Chronic liver disease with portal hypertension (porto-caval pressure gradient ≥5–≤10mmHg, including patients with TIPS)
- Previous radiotherapy or chemo-radiation ≥50Gy (salvage oesophagectomy)

Alternatively, patients must have **at least two of the following risk factors**:

- Arteriosclerosis score 2 according to van Rossum et al. [17] (aorta and coeliac axis)
- Malnutrition (BMI ≤ 18.5kg/m^2^)
- Obesity (BMI ≥ 35kg/m^2^)
- Heart failure with preserved ejection fraction (LVEF >55%)
- Active or former smoking
- Age > 65 years
- ECOG/WHO/Zubrodt PS score > 1
- chronic kidney disease stage 2–3 (GFR 30–89 ml/min/1.73 m^2^)
- chronic liver disease without portal hypertension (porto-caval pressure gradient ≤5mmHg)

**Patients with fulfilling any of the following criteria will be excluded from the study:** 

- Inability to provide informed consent or adhere to the study protocol, e.g. due to language problems, psychological disorders, or dementia.
- Patients younger than 18 years
- Patients scheduled for other technical variants of oesophagectomy, such as open, hybrid, or transhiatal procedures (intraoperative conversions to open access surgery will not be excluded)
- Chronic liver disease with portal hypertension (porto-caval pressure gradient >10mmHg)

### 2.6. Randomization, allocation and blinding

Randomization will be conducted with the use of an online randomization software (*http://randomizer.at*) one day prior to the scheduled surgical operation. The allocation ratio will be 1:1 and recorded in the software. The principle investigator or junior investigator will be responsible to generate the random allocation, enrolment and assignment of participants to interventions. Due to the nature of the intervention, blinding of participants and treating persons will not be feasible.

### 2.7. Methods of minimising bias

#### 2.7.1. Minimising selection bias

Consecutively screened and eligible patients will be included in the present study. A sufficient number of individuals will be recruited according to the sample size calculation (phase 2) in order to prevent random error and to achieve sufficient statistical power.

#### 2.7.2. Minimising performance bias

All esophagectomies will be performed in high-volume centres performing >20 minimally invasive esophagectomies per year [18]. Furthermore, the procedures will be performed by surgeons with a minimum case experience exceeding 100 oesophagectomies and pENP will exclusively be performed by interventional gastroenterologists or surgeons trained in ENP.

#### 2.7.3. Minimizing attrition bias

The trial will be reported according to the CONSORT statement [*www.consort-statement.org*]. The trial has been registered with *ClinicalTrials.gov* on 14.11.2019 with the unique identifying number NCT04162860 (*https://clinicaltrials.gov/ct2/show/NCT04162860*) and the Swiss National Clinical Trials Portal (SNCTP000003524). The trial protocol, including detailed description of clinical endpoints and statistical analysis plan will be published according to the SPIRIT statement [*www.spirit-statement.org*] to avoid risk of selective reporting.

#### 2.7.4. Other bias

Financial relationships with providers of medical devices or any conflict of interest that could inappropriately influence the work within this project will be stated explicitly [19]. The study design was conceived without any influence from the industry.

### 2.8. Trial organization

The principal investigator and the co-investigators are responsible for the preparation of the study protocol and the case report form (CRF). The principal investigator and the co-investigators are responsible for screening, recruitment, data collection and completion of the CRFs.

### 2.9. Interventions

#### 2.9.1. Explanation for the choice of comparators

It is not the current standard of practice to perform pENP during ttMILE in high-risk patients.

#### 2.9.2. Control Intervention

Patients in the control group will undergo a standard ttMILE without pENP. After 4–6 days, patency of the anastomosis will be assessed via either endoscopy, contrast radiography, or CT-scan, and results will be documented with a standard protocol.

#### 2.9.3. Experimental Intervention

Patients in the intervention group will undergo a standard ttMILE with pENP. After completion of the oesophago-gastric anastomosis, an Eso-SPONGE^®^ size 1 (overtube inner diameter 13mm, outer diameter 17mm) will be inserted via an intraoperative gastroscopy. pENP will be applied after completion of the oesophago-gastrostomy, but no later than 12 hours following the surgical intervention. The Eso-SPONGE^®^ device consists of an open-pored polyurethane foam fitted to a gastric tube. Postoperatively, secretions are continuously evacuated using a suction pump generating a negative pressure between 75–100 mmHg. The sponge is positioned under gastroscopic guidance at the level of the esophago-gastrostomy. ENP will remain in situ for 4–6 days and will be monitored based on clinical parameters (suction rate, vacuum leak rate, routine laboratory values, repeated routine chest x-rays). Discontinuation of ENP requires endoscopic removal of the Eso-SPONGE. During ENP removal, the anastomosis will be surveyed and documented with a standard protocol.

### 2.10. Outcomes

#### 2.10.1. Primary outcome pilot study

##### Feasibility

Feasibility will be assessed by assessing the number of eligible patients within the target population, recruitment rates, study refusal rates, study retention and follow-up rates as the participants move through the pilot study. Additionally, adherence rates to study procedures, intervention attendance, and engagement will be taken into consideration.

##### Safety

Any event of sponge-displacement, dislocation or intolerability will be recorded up to the 90-day postoperative follow-up period. Morbidity will be assessed using the Clavien-Dindo (CD) Classification [20] of postoperative complications, as well as the Comprehensive Complication Index (CCI) [21]. During the 90-day follow up period, the anastomotic stricture rate as a possible, anticipated adverse event of the ENP therapy will be recorded. Finally, the 90-day hospital readmission rate and mortality will be assessed.

#### 2.10.2. Primary outcome of the formal preSponge RCT

The primary outcome of the formal preSponge RCT will be postoperative length of hospitalisation. Patient discharge will be determined according to the subsequent, published, fit-for-discharge criteria [22].

- The patients’ oral/enteral nutritional requirements are met by oral intake of at least liquids with optional supplementary nutrition via jejunal feeding tube.
- The patient should have passed flatus.
- The patient does not require oxygen during mobilisation (short walk or climbing stairs) or at rest.
- Central venous catheters should be removed before discharge (unless present preoperatively).
- Adequate analgesia at rest and during mobilization (pain score <4 on a scale from 0 to 10) is achieved using both oral opioid and non-opioid analgesics.
- All vital signs should be normal unless abnormal preoperatively.
- Inflammatory parameters (white cell count, C-reactive protein) should be trending down and close to normal.
- There should be adequate support after discharge (assistance by family, ambulatory nursing, or rehabilitation facility).
- The patient does not experience clinical deterioration precluding discharge having already achieved “fit-for-discharge” status.

#### 2.10.3. Secondary Outcomes

Secondary outcome measures include morbidity and mortality assessed by the CD Classification [20] and the CCI during the 90-day postoperative follow-up period. [21] Additionally, AL rate and grade, conduit necrosis, infections, wound healing problems, length of intensive care unit stay, hospital readmissions, stricture will be assessed during the aforementioned follow up period. No follow-up later than 90 days is planned.

### 2.11. Data management

Data will be entered in a CRF by the principal investigator or a designated surgical resident. For data and query management, monitoring, reporting and coding an online web-tool (*https://www.esoHub.org*/) that meets FDA standards will be used for this study. It is the responsibility of the investigator to assure that all data in the course of the study will be entered completely and correctly in the respective database. Corrections to the CRF may only be made by the investigator or by other authorized persons. In case of corrections, the original data entries will be archived in the system and can be made visible. For all data entries and corrections, the date, time of day and name of the individual performing the entries will be recorded automatically. All study data will be archived for a minimum of 10 years after study completion or premature termination of the clinical trial. All data will be stored on the server of the “Klinik für Viszeral- und Transplantationschirurgie, Universitätsklinik Zürich”.

### 2.12. Monitoring

An external committee will monitor the study. All original data including all patient files, progress notes and copies of laboratory and medical test results will be available for monitoring. The study monitors will review 20–30% of the eCRFs and written informed consents. The documentation of each site visit will be submitted to the ethics committee. The accuracy of the data will be verified by reviewing the above referenced documents. The investigator’s site will collaborate with the Clinical Trials Centre (CTC) of the University Hospital Zurich to ensure monitoring.

### 2.13. Safety and reporting of serious adverse events

Device deficiencies and all adverse events (AE) including all serious adverse events (SAE) are collected, fully investigated and documented in the source document and appropriate CRF during the entire study period, i.e. from patient’s informed consent until the last protocol-specific procedure, including a safety follow-up period. AE/SAEs will be reported to the principal investigator within 24 h of being noted. If the principal investigator considers a SAE as unexpected and related to the study intervention, they will submit a report to the ethics committee within three days. Documentation includes dates of event, treatment, resolution, assessment of seriousness and causal relationship to device and/or study procedure. In case of relevant differences between the morbidity and the SAEs between the groups, a report will be submitted to the local ethics committee. The trial may be terminated based on the decision of the principal investigator according to the assessment of the ethics committee.

### 2.14. Statistical analysis

Basic demographic, pre-, intra-, and postoperative, as well as follow-up data will be compared separately for each randomised group. Firstly, patients will be analysed according to intention-to-treat and secondly, per protocol. The potential for bias due to lack of adherence to the study protocol is of importance. A study-independent statistician will perform the intended primary statistical analysis after completion of the study.

The primary endpoint (length of hospital stay until “fit for discharge” criteria are reached) typically shows a skewed distribution and will be compared between the two groups with a Mann-Whitney U test. As for the secondary outcomes, the two groups will be compared according to the CD classification using the Pearson chi-square test [20,23]. The CCI will be compared between the two randomized groups using the Student t test [21]. AL- and mortality rate will be compared using Fisher’s exact test. A two-sided P value < 0.05 will be considered significant.

### 2.15. Protocol version

This refers to the second version of the full study protocol from 08.07.2019. Protocol modifications will be reported to all investigators, the local ethics committee, the Clinical Trials Register, all trial participants, and the journal.

## 3. Discussion

This prospective multi-centre, parallel-group RCT aims to assess the effect of pENP on AL after ttMILE in high-risk patients. We hypothesise that patients at increased risk for AL undergoing pENP suffer from fewer postoperative complications and therefore have a reduced length of hospital stay and reach “fit for discharge” criteria faster. Until now, endoscopic placement of the polyurethane foam at the level of the anastomosis with ENP has been exclusively used for the treatment of AL, whilst published data regarding its prophylactic application remains scarce [11,12,13,24,25]. In an experimental pilot study by Scott et al. on pigs undergoing Ivor Lewis esophagectomy, experimental anastomotic defects were treated with pENP or received no specific treatment. Half of the pigs in the control group died within 24 hours and were excluded from analysis, whereas the surviving controls showed frank AL and pleural contamination with gastric contents. In contrast, no leaks were detected in the ENP cohort after 3–7 days follow-up (p = 0.03). This study demonstrated that pENP may have the potential to close leaks that otherwise would persist without surgical or endoscopic intervention [26]. Further evidence supporting this hypothesis stems from our own Institution’s retrospective case-series of 20 consecutive patients undergoing minimally invasive oesophagectomy with pENP, anastomotic healing was uneventful in 19/20 anastomoses resulting in an AL rate of 5%. One contained AL healed without further complications following a second course of ENP. There were no adverse events attributable to pENP therapy [15].

The present study is the first randomised controlled trial that assesses the possible benefits of pENP therapy after ttMILE. Whilst the benefit of pENP has to be evaluated in a first step, another important question is the identification of the patient cohort that could benefit from this intervention. We believe that pENP could be especially beneficial in patients with multiple comorbidities who are at high risk for AL as defined by our inclusion criteria. The primary endpoint of the study (length of hospital stay until “fit for discharge”) was chosen to reflect a potentially faster short-term recovery. Although AL rate is an important secondary endpoint that may be reduced with pENP, our experience shows that more importantly, the course of AL occurring during pENP is remarkably more benign without septic complications. Therefore, pENP may seal potential full-thickness defects at an early stage thereby preventing free AL. If the results of the study are positive, the findings could potentially improve the postoperative management of patients undergoing oesophagectomy.

## 4. Ethics and Dissemination

The ethics committee of the University of Zurich reviewed and approved this study protocol on 11.11.2019 (BASEC-Nr: 2019-00562). Secondary approval of the corresponding ethical bodies of all other participating centres will be obtained. To ensure patient’s rights and safety, the responsible investigator will ensure that the trial will be conducted according to the ethical principles laid out in the declaration of Helsinki. [27] Before participation in the trial, written informed consent will be obtained from each study participant. The trial protocol has been formulated in accordance with the recommendations of the Consolidated Standards of Reporting Trials (CONSORT) and SPIRIT guidelines [28,29]. The SPIRIT checklist is provided as additional file 1.

The results of the study will be presented at national and international congresses on corresponding fields of interest. Written publication of the results is planned within a peer-reviewed surgical journal. The authorship for written publications has to be confirmed by all lead investigators and will only be granted in the case of substantive contributions to the design, conduct, data analysis, and interpretation. After completion of the full study report, anonymized participant-level datasets and the statistical code for generating results will be available by contacting the principal investigator.

## Abbreviations

AE: Adverse events

AL: Anastomotic leakage

ASA: American Society of Anesthesiologists Classification

BMI: Body mass index

CCI: Comprehensive Complication Index

CRF: Case report form

CTC: Clinical Trials Centre

ECCG: Esophagectomy Complications Consensus Group

ENP: Endoluminal negative pressure

FEV1: Forced expiratory volume in one second

FVC: Forced vital capacity

GFR: Glomerular filtration rate

LVEF: Left ventricular ejection fraction

pENP: Prophylactic endoluminal negative pressure

RCT: Randomised controlled trial

SAE: Serious adverse events

TIPS: Transjugular intrahepatic portosystemic shunt
